# Supplementary material for: End Sequence Analysis Toolkit (ESAT) expands the extractable information from single-cell RNA-seq data
Source: Genome Res. 2016 Oct;26(10):1397–410. doi: 10.1101/gr.207902.116 (PMC5052061; doi:10.1101/gr.207902.116)
Supplement: Supplemental Material [file supp_gr.207902.116_Supp_Materials.tar.gz › ESAT-esat_v0.1/doc/html/classbroad_1_1core_1_1sequence_1_1_transcribed_sequence_composition-members.html]

ESAT: Member List


ESAT


- Main Page
- Related Pages
- Packages
- Classes
- Files

- Class List
- Class Index
- Class Hierarchy
- Class Members

All Classes Namespaces Files Functions Variables Enumerator Pages

- broad
- core
- sequence
- TranscribedSequenceComposition

broad.core.sequence.TranscribedSequenceComposition Member List

This is the complete list of members for broad.core.sequence.TranscribedSequenceComposition, including all inherited members.

|  |  |  |
| --- | --- | --- |
| allNucleotides | broad.core.sequence.TranscribedSequenceComposition | protected |
| getTranscribedBaseAtBeginningPosition(Gene parent, Alignment read, int offset) | broad.core.sequence.TranscribedSequenceComposition |  |
| main(String[] args) | broad.core.sequence.TranscribedSequenceComposition | static |
| TranscribedSequenceComposition(String bamFile, String geneBedFile, String genomeFasta, TranscriptionRead transcriptionRead) | broad.core.sequence.TranscribedSequenceComposition |  |
| TranscribedSequenceComposition(String bamFile, String geneBedFile, String bedFileRegionsOfInterest, String genomeFasta, TranscriptionRead transcriptionRead) | broad.core.sequence.TranscribedSequenceComposition |  |


---

Generated on Fri Aug 15 2014 15:10:56 for ESAT by  

 1.8.7
